# The role of SARS‐CoV‐2 target ACE2 in cardiovascular diseases

**DOI:** 10.1111/jcmm.16239

**Published:** 2021-01-14

**Authors:** Hanzhao Zhu, Liyun Zhang, Yubo Ma, Mengen Zhai, Lin Xia, Jincheng Liu, Shiqiang Yu, Weixun Duan

**Affiliations:** ^1^ Department of Cardiovascular Surgery The First Affiliated Hospital The Air Force Medical University Xi’an China; ^2^ Department of Dermatology and Venereology Peking University First Hospita Beijing China

**Keywords:** ACE2, cardiovascular diseases, RAS, SARS‐CoV‐2

## Abstract

SARS‐CoV‐2, the virus responsible for the global coronavirus disease (COVID‐19) pandemic, attacks multiple organs of the human body by binding to angiotensin‐converting enzyme 2 (ACE2) to enter cells. More than 20 million people have already been infected by the virus. ACE2 is not only a functional receptor of COVID‐19 but also an important endogenous antagonist of the renin‐angiotensin system (RAS). A large number of studies have shown that ACE2 can reverse myocardial injury in various cardiovascular diseases (CVDs) as well as is exert anti‐inflammatory, antioxidant, anti‐apoptotic and anticardiomyocyte fibrosis effects by regulating transforming growth factor beta, mitogen‐activated protein kinases, calcium ions in cells and other major pathways. The ACE2/angiotensin‐(1‐7)/Mas receptor axis plays a decisive role in the cardiovascular system to combat the negative effects of the ACE/angiotensin II/angiotensin II type 1 receptor axis. However, the underlying mechanism of ACE2 in cardiac protection remains unclear. Some approaches for enhancing ACE2 expression in CVDs have been suggested, which may provide targets for the development of novel clinical therapies. In this review, we aimed to identify and summarize the role of ACE2 in CVDs.

## 1 INTRODUCTION

**FIGURE 1:**
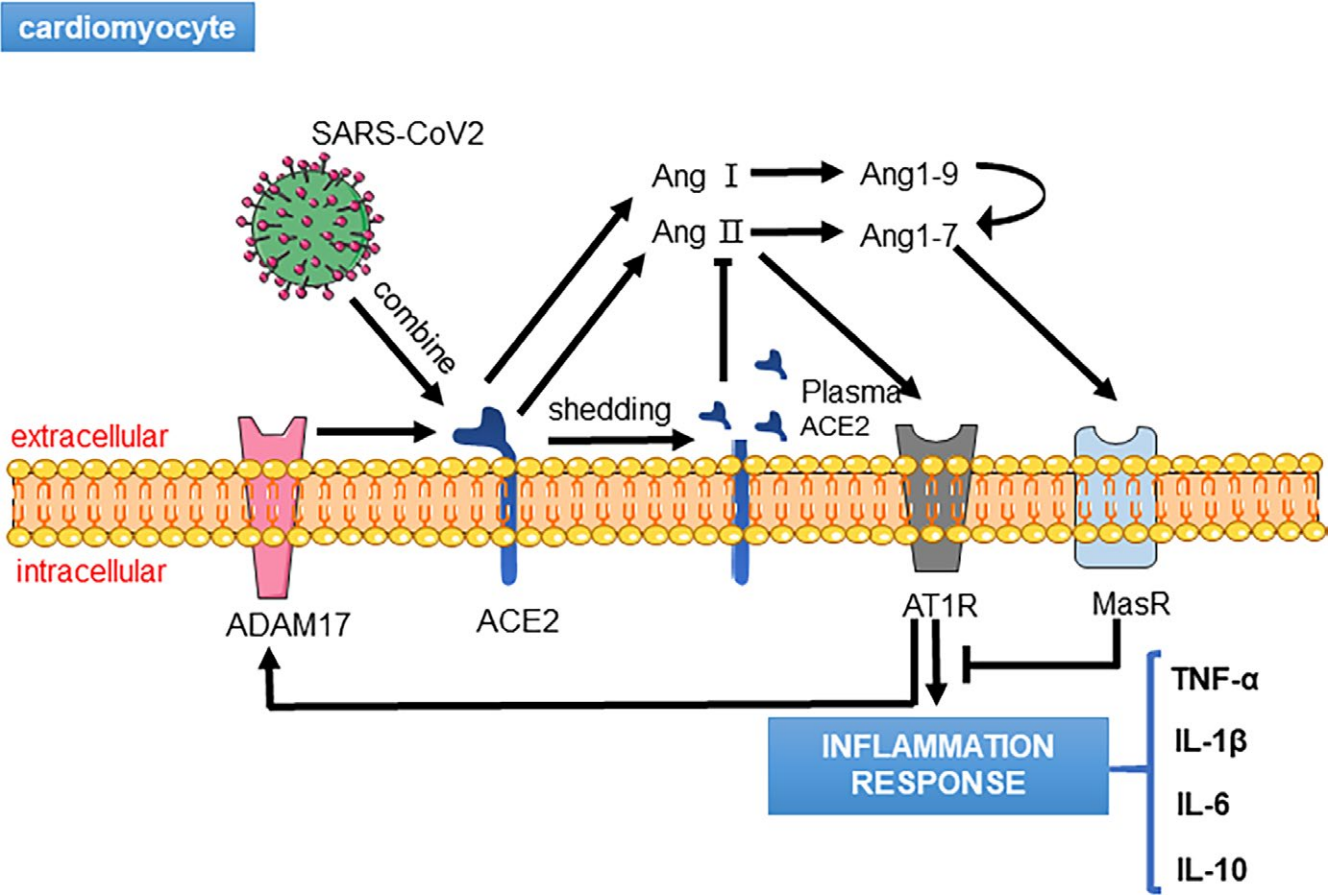
Severe acute respiratory syndrome coronavirus 2 (SARS‐CoV‐2) binds to angiotensin‐converting enzyme 2 (ACE2) and induces ACE2 shedding to produce soluble ACE2 in serum, which triggers an angiotensin II (Ang II)‐mediated inflammation response

In December 2019, a novel coronavirus‐induced pneumonia called novel coronavirus disease 2019 (COVID‐19) first appeared in Wuhan, China. Due to the spread of COVID‐19, more than 200 countries around the world have been impacted. As of the end of September 2020, there have been more than 20 million confirmed cases of COVID‐19, and over 900,000 patients have died from the disease.^1^ Based on epidemiological analyses, COVID‐19 patients suffer from severe multiple organ injuries, including the lungs, kidneys and liver, which are also closely related to adverse outcome in cardiovascular diseases (CVDs).^2^, ^3^, ^4^, ^5^ Due to the high homology between severe acute respiratory syndrome coronavirus (SARS‐CoV) and severe acute respiratory syndrome coronavirus 2 (SARS‐CoV‐2), several studies have demonstrated the mechanism by which these viruses invade the human body. SARS‐CoV‐2 invades cells by relying on the spike protein of its surface, which is similar to SARS‐CoV. The S1 subunit of the spike protein can bind to angiotensin‐converting enzyme 2 (ACE2) of the cell membrane and form a complex, which allows the virus to enter the cell by endocytosis and thereby induce cellular damage (Figure 1).^6^, ^7^ Because of the distribution and the function of ACE2, it must play a decisive role in COVID‐19 patients with multiple organ damage.

ACE2 was first discovered in 2000 by Donoghue and Tipnis.^8^, ^9^ The human ACE2 gene is 40 kb long, located on chromosome Xp22 and composed of 18 exons.^8^, ^9^ The protein of human ACE2, which is a type I integral membrane glycoprotein with an N‐terminal signal peptide region, a hydrophobic region near the C terminus and a single active site catalytic region, is approximately 120 kD and consists of 805 amino acids.^9^ ACE2 has 42% homology with the amino acid sequence of ACE and is widely expressed in the body, including the heart, vasculature, kidneys, testes, gastrointestinal tract, brain and lungs.^10^, ^11^, ^12^ Moreover, ACE2 is not only a plasma membrane‐bound ectoenzyme; its soluble active form exists in plasma and urine, and it can be detected via routine blood tests.^13^

In the heart, ACE2 is mainly expressed in cardiomyocytes, cardiac fibroblasts and coronary artery endothelial cells and also serves as an important endogenous antagonist of the renin‐angiotensin system (RAS), which mainly converts angiotensin II (Ang II) to angiotensin‐(1‐7) [Ang‐(1‐7)] and metabolizes angiotensin I (Ang I) to generate angiotensin‐(1‐9) [Ang‐(1‐9)]. Ang‐(1‐7) and Ang‐(1‐9) have been proven to have significant beneficial effects on the cardiovascular system.^9^, ^14^ Ang‐(1‐7) generally binds to the Mas receptor (MasR), a G protein‐coupled receptor discovered in 1986, and further regulates downstream molecular pathways, including the mitogen‐activated protein kinase (MAPK), protein kinase B (AKT) and oxidative stress‐related pathways.^15^, ^16^, ^17^, ^18^ ACE2 restrains Ang II accumulation and down‐regulates the angiotensin II type 1 receptor (AT1R), such that the ACE2/Ang‐(1‐7)/MasR axis has the opposite effect of the ACE/Ang II/AT1R axis and promotes anticardiovascular remodelling and mediate vasodilation.^19^ In addition, angiotensin II type 2 receptor (AT2R), one of the core receptors of the angiotensin family, is also activated by Ang‐(1‐7).^20^ ACE2/Ang‐(1‐7)/AT2R axis has the same beneficial effects as ACE2/Ang‐(1‐7)/MasR axis; however, the researches about the axis are limited.^21^

Given the existing knowledge of ACE2 distribution and function in the heart, we hypothesized that ACE2 plays a crucial role in the progression of SARS‐CoV‐2‐induced myocardial injury. The present review aims to identify and summarize the functions and mechanisms of ACE2 in clinical and animal models of major CVDs.

## 2 COVID‐19, ACE2 AND CVDS

Due to the high expression of ACE2 in the heart and the mechanism of SARS‐CoV‐2 transfection, we speculate that the poor prognosis of COVID‐19 patients may be correlated with heart injury. At present, there are two major hypotheses about SARS‐CoV‐2‐mediated damage to the heart. On the one hand, SARS‐CoV‐2 directly causes heart injury in patients without any CVDs; on the other hand, SARS‐CoV‐2 can also contribute to the negative prognoses of patients with heart diseases.^22^, ^23^ In addition, some severe COVID‐19 patients display higher levels of cardiac troponin I (cTnI), N‐terminal pro‐B‐type natriuretic peptide (NT‐proBNP) and creatine kinase‐MB (CK‐MB), which collectively demonstrates that SARS‐CoV‐2 aggravates heart injury.^24^, ^25^, ^26^, ^27^ A meta‐analysis of cTnI in COVID‐19 patients including four studies exhibited that the standardized mean difference (SMD) of cTnI was 25.6 ng/L.^26^ In addition, a multi‐centre cohort study involving 191 patients infected with SARS‐CoV‐2 showed that of the 54 deceased patients, 48% had hypertension, 24% had coronary heart disease, and approximately 23% had heart failure (HF).^28^ Based on the clinical phenomena of ACE2 and heart injury, several studies have suggested that SARS‐CoV‐2 binds to ACE2 to enter cells and increases the expression levels of disintegrin and metalloproteinase 17 (ADAM17), which, in turn, induce ACE2 shedding. Thus, the function of ACE2 is compromised and simultaneously accompanied by increasing ACE and Ang II expression levels. A higher level of Ang II not only has severe negative effects on the heart, but also increases cytokine release, including interleukin (IL)‐6 and IL‐7, which activate the MAPK pathway and thereby increase ADAM17 expression to form a positive feedback loop. Thus, ACE2 appears to be a major regulating factor in COVID‐19 patients.^29^, ^30^, ^31^ In recent years, an increasing number of studies have confirmed that neither ACE inhibitors (ACEIs) nor Ang II receptor blockers (ARBs) are associated with high mortality, which suggests that ACEIs and ARBs cannot exacerbate the prognoses of COVID‐19 patients. Meanwhile, there has been no evidence to support that evaluating ACE2 expression can improve heart injury during SARS‐CoV‐2 infection.^32^, ^33^, ^34^ In addition, although some drugs, like remdesivir, lopinavir and dexamethasone, have been identified to improve COVID‐19 patients’ symptoms and attenuate inflammatory response, there are no specific drugs invented to treat cardiovascular injury caused by SARS‐CoV‐2. ^35^, ^36^, ^37^

Based on the aforementioned information, we believe that the most powerful approach of addressing SARS‐CoV‐2‐induced heart injury is to clarify the upstream and downstream molecular pathways of ACE2 in CVDs and find effective strategies to protect the heart.

## 3 ACE2 AND MYOCARDIAL INFARCTION (MI)

MI has a high incidence among patients with CVDs, and in the United States, nearly 0.8 million people are reported to suffer from MI each year.^38^ In recent years, multiple studies have shown that the RAS, especially ACE2, is involved in MI‐induced myocardial remodelling.^39^ In the early stage of MI, both ACE2 and ACE levels are remarkably increased in the heart, whereas in the late stage, ACE2 expression declines and is accompanied by HF, indicating the role of ACE2 against the RAS.^40^, ^41^ Similarly, clinical research has found that the serum ACE2 level of MI patients is significantly higher than that of healthy individuals and results in a negative prognosis.^42^, ^43^ Further, the results indicate that the serum level of ACE2 may be a candidate for identifying the degree of myocardial injury. Inflammatory infiltration and myocardial fibrosis are the two major factors that induce cardiac structure remodelling during MI. Following developments in basic research, ACE2 is now believed to be resistant to the negative effect of ACE on myocardial remodelling post‐MI mainly depending on the ACE2/Ang‐(1‐7)/MasR axis. In MI, loss of the ACE2 gene leads to ventricular remodelling, increased myocardial fibrosis, neutrophilic infiltration and superoxide production via up‐regulation of matrix metalloproteinase (MMP) 2, MMP9, interferon‐γ, IL‐6 and chemokines as well as regulation of phosphorylation of the extracellular regulated protein kinase (ERK) 1/2 and c‐Jun N‐terminal kinase (JNK) 1/2 signalling pathways.^44^ In contrast, overexpression of ACE2 can reverse collagen deposition by inhibiting the transforming growth factor‐β (TGF‐β) pathway with decreasing collagen Ⅰ and Ⅲ, which also reduces the expression levels of inflammation‐related factors ACE and Ang Ⅱ.^45^, ^46^, ^47^ Diminazene (DIZE), an activator of ACE2, is also used in some studies to improve myocardial function following MI. Chen et al illustrated that DIZE attenuated MI in rats via a novel signalling pathway mediated by ACE2/AT1R/MasR.^48^ Given these findings, ACE2 may also be a therapeutic target for MI; however, as no effective drugs can adequately evaluate ACE2 expression in the heart, the concrete molecular mechanisms underlying ACE2 actions need to be further investigated.

## 4 ACE2 AND HYPERTENSION

Hypertension is one of the most common CVDs that is characterized by vascular remodelling and endothelial injury and leads to severe prognosis. RAS is a participant in the development of the disease.^49^ It is known that the ACE/Ang Ⅱ/AT1R axis regulates the constriction of blood vessels, and most available drugs aim to block this pathway. The ACE2/Ang‐(1‐7)/MasR axis is a potential target for combating the negative effects of the ACE/Ang Ⅱ/AT1R axis on hypertension, and ACE2 plays a key role in this axis. ACE2 regulates blood pressure under physiological conditions, and down‐regulation of ACE2 gene leads to significant increase in the blood pressure along with an excess accumulation of Ang Ⅱ.^50^ In addition, ACE2 is significantly decreased in spontaneously hypertensive rat (SHR) or Ang Ⅱ‐induced hypertension models.^51^, ^52^ Interestingly, ACE2 and Ang Ⅱ can regulate each other to maintain a balance. Ang Ⅱ up‐regulates AT1R and increases ADAM17 expression, which causes ACE2 shedding and diminishes the protective impact of ACE2 in hypertension whereas Ang Ⅱ can be converted into Ang‐(1‐7) by ACE2 to inhibit its own negative effect.^53^, ^54^ Rentzsch et al found that Ang‐(1‐7), which directly regulates blood pressure and improves endothelial function via activating the ACE2 gene, is the major downstream regulator of ACE2.^55^ However, aside from Ang‐(1‐7), the downstream mechanism of ACE2 in hypertension remains unclear. Several studies have determined that overexpression of ACE2 in hypertension increases AT2R and MasR expression and inhibits AT1R expression.^56^, ^57^ Furthermore, they evaluated nitric oxide (NO) release and reported down‐regulation of inflammation‐related pathways mediated by IL‐1 b, IL‐6, TNF‐a and NF‐kB.^56^, ^57^ Activation and modification of ACE2 are essential for development of hypertension. DIZE and fibroblast growth factor 21 (FGF21) can up‐regulate ACE2 expression to improve pulmonary hypertension and decrease inflammation‐induced endothelial cell injury.^58^, ^59^ Zhang et al found that AMP‐activated protein kinase (AMPK) phosphorylated ACE2 Ser680 in endothelial cells can enhance the function of ACE2 depending on regulation of Ang‐(1‐7) and nitric oxide synthase.^60^ From the aforementioned information, it is clear that there is limited knowledge on known signalling pathways of ACE2, and hence, further studies are warranted to analyse the potential targets in hypertension. Recently, it has been found that ACE2 overexpression also protects against neurogenic hypertension via regulation of baroreflex and autonomic function in the central nervous system (CNS).^61^ Although the role of ACE2 in hypertension is clear, the underlying mechanism needs to be elucidated to identify the relevant downstream signalling pathways and clinical trials need to be designed to determine whether up‐regulating the ACE2 level can lower blood pressure in patients.

## 5 ACE2 AND ARRHYTHMIA

Arrhythmia is myocardial disease that is caused by electrophysiological dysfunction and is often associated with oxidative stress.^62^ The RAS has been proven to be involved in the development of arrhythmia.^63^ As a significant protein in the RAS, ACE2 exerts negative effects on arrhythmia during the early stage. In ACE2 transgenic hearts, the gap junction proteins connexin40 and connexin43 were significantly down‐regulated, thus prolonging PR and QRS durations and potentially inducing conduction disturbances and lethal ventricular arrhythmias.^64^ However, recent studies have shown that ACE2 may be an important protective factor in lethal arrhythmia. Overexpression of ACE2 in an atrial fibrillation model activated extracellular signal‐regulated kinases and up‐regulated MAPK levels, as well as induced a decrease in the level of MAPK phosphatase 1 (MKP‐1).^65^ These findings resulted from decreases in atrial fibrosis collagen protein markers and TGF‐β, which may be one of the molecular mechanisms underlying the protective effect of ACE2 in atrial fibrillation.^66^ In addition, strong evidence has shown that ACE2 agonist DIZE can reverse hyperglycaemia‐induced cardiac electrical changes in ventricular repolarization, thereby shortening the QT and QTc intervals on an electrocardiogram.^67^ Moreover, Ang‐(1‐7) is a crucial protein in the downstream regulation of ACE2 that also plays a role in antiarrhythmic effects via reducing action potential repolarization phases and decreasing the late sodium (Na^+^), L‐type Ca^2+^ and Na^+^‐Ca^2+^ exchanger currents, thereby further mediating the balance of intracellular Ca^2+^ and sarcoplasmic reticulum Ca^2+^.^68^, ^69^ Therefore, additional emphasis should be placed on the role of ACE2 in arrhythmia, and new strategies for clinical antiarrhythmic drugs should be developed.

## 6 ACE2 AND DIABETES RELEVANT CVDS

Metabolic dysfunction is a major consequence of diabetes and includes oxidative stress, inflammation and multiple organ injuries in the later stage, especially to the heart. The specific pathogenesis is thought to be related to the RAS.^70^ Recent studies have shown that the ACE2/Ang‐(1‐7)/MasR axis plays a significant role in diabetes‐induced cardiomyopathy. The expression of ACE2 decreased in the myocardial tissue of diabetic rats; furthermore, it inhibited myocardial collagen expression as well as promoted collagen degradation by regulating the TGF‐β pathway and activating MMP2.^71^ Interestingly, ACE2 also exerts this role by regulating Ang‐(1‐7) and Ang‐(1‐9) before activating angiotensin II type 2 receptor (AT2R).^72^, ^73^, ^74^ From this point of view, TGF‐β and MMP2 may be downstream targets of AT2R. Moreover, administration of Ang‐(1‐7) significantly reduced myocardial lipid accumulation by up‐regulating the expression of myocardial triglyceride lipase, which occurred as a result of up‐regulating the level of sirtuin‐1 (SIRT1). The activation of SIRT1 further regulated transcriptional activity of FOXO1 via SIRT1‐mediated deacetylation, which was proved as one of the potential protective targets on oxidative stress and inflammatory response.^75^, ^76^, ^77^ In addition, Ang‐(1‐7) can activate the expression of sarcoplasmic reticulum Ca^2+^‐ATP enzyme to improve the left ventricular systolic dysfunction and right ventricular fibrosis caused by hyperglycaemia.^73^ Clinical investigations of drugs targeting the ACE2/Ang‐(1‐7)/MasR axis have proven that ARB drugs like azilsartan and statins like atorvastatin can delay the progression of diabetic cardiomyopathy by increasing the expression levels of ACE2 and Ang‐(1‐7) combined with ACEI or DIZE and neprilysin inhibition therapy to provide better heart protection.^78^, ^79^, ^80^, ^81^ Finally, from our review, there is no evidence that ACE2 has effect on regulating blood glucose to improve diabetes relevant CVDs. These data suggest that the ACE2/Ang‐(1‐7)/MasR axis may serve an important target for the treatment of diabetes‐induced myocardial injury in the future.

## 7 ACE2 AND OTHER CVDS‐INDUCED HF

HF is a terminal stage of heart injury caused by various factors and characterized by systolic dysfunction. Besides of the above diseases, dilated cardiomyopathy, age‐related myocardial damage and cardiac afterload pressure overload all can induce HF happening. No clinically effective treatment strategies or sensitive predictive indicators exist to provide an early warning of the disease.^82^ As ACE2 was discovered, an increasing number of studies have prioritized identifying a novel therapeutic breakthrough based on the ACE2 pathways. Some reports have focused on the serum level of ACE2 in HF patients, which revealed that serum ACE2 is increased in HF and indicated that ACE2 has the potential to become a reliable marker with the same efficacy as BNP.^83^, ^84^, ^85^ The main function of ACE2 in HF is the degradation of Ang Ⅱ whereas Ang‐(1‐7) combats oxidative stress, fibrosis and inflammation. In a pressure‐overload heart, a model for inducing dilated cardiomyopathy, Bodiga et al and Patel et al reported that loss of the ACE2 gene led to increased nicotinamide adenine dinucleotide phosphate (NADPH) oxidase activity and fibrosis, accompanied by up‐regulated NADPH oxidase 2 (NOX2), p47phox, MMP2 and MMP9. This in turn activated ERKs, signal transducers and activators of the transcription (STAT) and AKT pathways, thereby resulting in further cardiac dysfunction. However, these observations were significantly reversed by a supplemental Ang‐(1‐7) or AT1R blockade.^86^, ^87^, ^88^ In age‐dependent cardiomyopathy, ACE2 deficiency not only exacerbates oxidative stress injury but also stimulates the release of inflammatory factors via activating the MAPK pathway.^88^ As the absence of ACE2 causes severe myocardial damage, drugs that up‐regulate ACE2 expression may have a positive effect on improving cardiac function. Wang et al utilized common clinical Sartan drugs, including olmesartan, candesartan, telmisartan, losartan, valsartan and irbesartan, and found that only olmesartan and candesartan increased ACE2/Ang‐(1‐7)/MasR expression and resisted pressure overload‐induced pathological changes in the heart with markedly declined ACE and AT1R expression as well as inhibited ERK phosphorylation.^89^ In clinical research, spironolactone, a mineralocorticoid receptor blocker, significantly inhibited oxidative stress by lowering ACE activity and increasing ACE2 expression in the macrophages of congestive HF patients. Eplerenone also appeared to attenuate NADPH oxidation, leading to the same effect as spironolactone in macrophages treated with aldosterone.^90^ B38‐CAP, discovered from Paenibacillus sp, has a structure similar to ACE2 and significantly improves pressure overload‐induced HF and cardiac hypertrophy.^91^ Notably, recombinant human ACE2 (rhACE2) is also widely used in basic and clinical research for converting Ang Ⅱ to Ang‐(1‐7) against dilated cardiomyopathy in HF patients. RhACE2 has also been observed to attenuate doxorubicin‐induced cardiac dysfunction by protecting cardiomyocyte autophagy.^92^, ^93^ However, despite the increasing number of studies that have focused on the therapeutic role of ACE2 for patients with HF, specific target drugs need to be developed.

## 8 CONCLUSION

**FIGURE 2:**
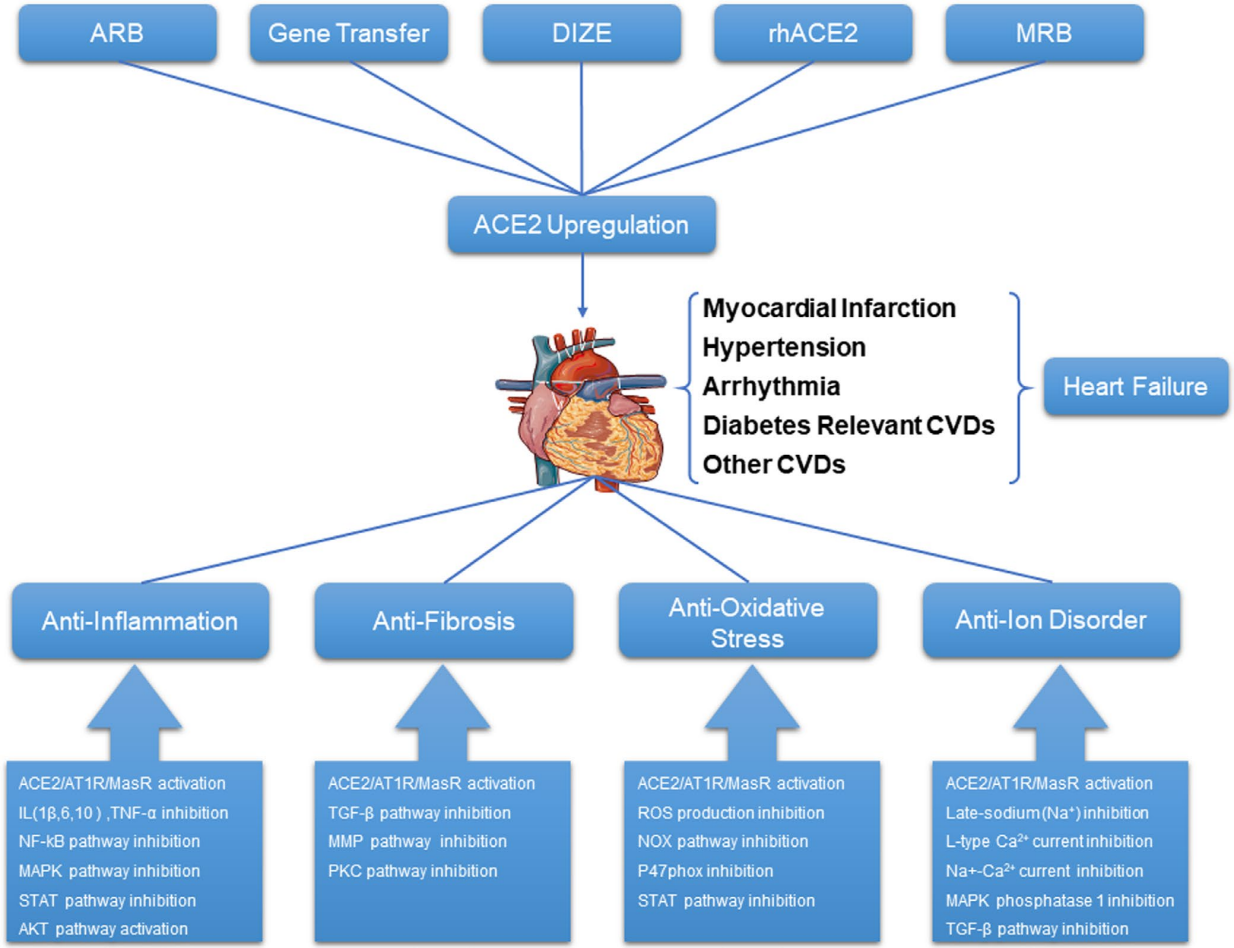
A diagram depicting the method for up‐regulating ACE2 expression and the downstream molecular mechanism of ACE2 in cardiovascular diseases (CVDs)

A significant amount of data has shown that ACE2 plays a crucial role in countering the development and progression of CVDs. In addition, the ACE2/Ang‐(1‐7)/MasR axis can improve CVDs through vasodilation, antiventricular remodelling, anti‐inflammatory, antioxidant and antimyocardial fibrosis effects. Studies on the other signalling pathways of ACE2 are scarce, and only a handful of classic pathways have been explored in CVDs. In hypertension, the function of ACE2 is to vasodilate and reduces blood pressure primarily by antagonizing the ACE/Ang II/AT1R axis, which can also affect the NO signal pathway in the CNS. In arrhythmias, ACE2 activity can reduce the occurrence of arrhythmic events and regulate calcium‐iron discrepancies. In addition, the antidiabetic cardiomyopathy effect of ACE2 is closely related to its antifibrosis effect via regulation of the TGF‐β signalling pathway. Furthermore, ACE2 can reduce myocardial collagen deposition induced by hyperglycaemia and activate MMP2. HF is the terminal state of cardiac dysfunction resulting from various factors, and up‐regulating ACE2 can reverse HF by improving cardiac remodelling, inhibiting oxidative stress and decreasing inflammation. The specific agonists of ACE2, DIZE and rhACE2 are recognized to have good efficacy in the treatment of CVDs (Figure 2).

Nowadays, the number of COVID‐19 patients is raising rapidly, and the relevant drugs are limited. Moreover, vaccine development such as inactivated virus, adenovirus‐vectored investigational vaccine and mRNA‐based vaccine, has been in clinical trial stage, but which needs further long‐term clinical experiments. As the receptor for SARS‐CoV‐2, ACE2 has become a major concern in recent months, and related investigations have shown that it is significantly related to the virulence and severity of the virus. Due to its high expression in the heart tissue as well as clinical studies that have identified widespread myocardial injury in infected patients, understanding the pathophysiological role of ACE2 in the heart is of the utmost importance. As a result, ACE2 is regarded as a potential target for the treatment of COVID‐19 infection. In the feature, we believe the best methods defending against cardiovascular injury induced by SARS‐CoV2 infection are to focus on vaccine development and medicines targeting ACE2. Thus, identifying mechanisms to inhibit the ACE2 binding site for SARS‐CoV‐2 without harming its normal physiological function in the heart presents a new challenge for researchers in the midst of the current global pandemic.

## CONFLICT OF INTEREST

The authors declare that there are no conflicts of interest.

## AUTHOR CONTRIBUTIONS

**Hanzhao Zhu:** Writing‐original draft (lead); Writing‐review & editing (equal). **Weixun Duan:** Funding acquisition (equal); Methodology (equal); Resources (equal); Supervision (lead); Writing‐review & editing (equal). **Shiqiang Yu:** Funding acquisition (lead); Resources (equal); Supervision (equal). **Liyun Zhang:** Writing‐original draft (equal); Writing‐review & editing (equal). **Yubo Ma:** Writing‐original draft (equal); Writing‐review & editing (equal). **Mengen Zhai:** Validation (equal); Visualization (equal). **Lin Xia:** Methodology (equal); Validation (equal). **Jincheng Liu:** Conceptualization (equal).
